# Unpacking the ‘black box’ of suicide: A latent class analysis predicting profiles of suicidal ideation in a longitudinal cohort of adolescent girls from India

**DOI:** 10.1371/journal.pgph.0003130

**Published:** 2024-05-08

**Authors:** Anushka R. Patel, Kelly E. Dixon, Abhijit Nadkarni

**Affiliations:** 1 Department of Epidemiology, Harvard School of Public Health, Boston, Massachusetts, United States of America; 2 Department of Psychology, University of Colorado at Colorado Springs, Colorado Springs, Colorado, United States of America; 3 Addictions and Related Research Group, Sangath, Goa, India; 4 Centre for Global Mental Health, Department of Population Health, London School of Hygiene and Tropical Medicine, London, United Kingdom; COMSATS University Islamabad, PAKISTAN

## Abstract

**Introduction:**

Indian women account for 37% of global suicide-related deaths. As suicide is a growing concern among adolescent girls, identifying the social determinants of suicide with this group targeted prevention. We selected social determinants that include intersectional identities and broader syndemics; we then used longitudinal data from a prospective cohort of adolescent girls from Northern India to classify them into unique profiles across multiple socioecological levels.

**Methods:**

Girls aged 10–19 (*N* = 11,864) completed self-report questionnaires measuring socio-demographic and trauma exposure variables. At three-year follow-up, they were asked to indicate current suicidal ideation (SI). We conducted latent class analysis (LCA) to classify profiles and then predicted risk of current SI at three-year follow-up.

**Results:**

LCA supported a four-class solution: a ‘privileged’ class (Class 1; *n* = 1,470), a ‘modal’ class (Class 2; *n* = 7,449), an ‘intergenerational violence’ class (Class 3; *n* = 2,113), and a ‘psychological distress’ class (Class 4; *n* = 732). Classes significantly predicted odds ratios (OR) for SI at follow up; women in Class 4 were associated with the greatest likelihood of SI (OR 1.84, 95% CI 1.38, 2.47), suggesting that psychological distress factors confer greatest risk.

**Conclusion:**

Results of the distinct classes of risk and protective factors indicate targets for policy-level interventions. Disrupting cycles of psychological distress and substance use, increasing access to behavioral interventions, and intervening to mitigate intergenerational violence may be particularly impactful with this population.

## Introduction

Indian women account for 37% of suicide-related deaths among women worldwide [1]. Suicide is the leading cause of death among young Indians, causing 73% of deaths in the 15–29 age category [1]. The unique stressors they face in adolescence underscores the gendered nature of risks for suicide; these social determinants include socioeconomic factors (e.g., poverty, employment status [2, 3], violence and trauma [4, 5], and discrimination [6, 7]). All these factors also increase the risk of mental illness, such as depression and post-traumatic stress disorder [8], which amplifies the risk of suicide. Yet, in low-and-middle-income countries (LMIC), only a quarter of the variance in suicidal outcomes is explained by mental health diagnoses; instead, the broader structural conditions might play a greater role [9].

Suicide is caused by a complex and dynamic interplay of many factors related to the individual and their social environment across the lifespan. At an individual level, intersectional identities (e.g., age, income, and mental health) [10–12] confer risk for suicide. At the interpersonal and communal levels, social determinants converge to create concentrated conditions of adversity or *syndemics* [13] (e.g., poverty, unemployment, addiction, and violence) that also confer risk for suicide. Therefore, efforts to prevent suicide require an intersectional lens towards individuals’ identities in concert with the broader social ecology they navigate.

Gender itself is an important determinant of suicide [14], particularly in cultural settings where gender roles are strict and movement away from gender expectations is socially punished. Examples of social stressors that contribute to suicide attempts in Indian women include marital conflict, husband’s infidelity, and domestic violence [9, 12, 15]. As such, marriage is a risk factor for women and 57% of all completed suicides between 1990–2016 were by housewives [1]. The social role transition for women, which happens rapidly during puberty, also partly explains the how these stressors contribute to the risk of suicide.

The onset of adolescence catalyzes physical and psychological changes that interface with varied and complex stressors. At an individual level, adolescence is a high-risk developmental period because people are most likely to experience their first depressive episode during this time [16]. At an interpersonal level, significant changes in peer relationships occur during adolescence and peer groups become more influential than parents in shaping behaviors; these changes can contribute to tensions within the home environment. At the community level, girls’ social roles and gender expectations become more focused towards their ‘marriageability’–that is, education takes a backseat, especially in rural areas of India [17]. As marriage is often arranged by parents and dating is generally taboo, girls are often unprepared for stressors related to marriage.

As risk for suicide is notoriously complex to predict(18), intersectional identities *and* synergistic social determinants (i.e., syndemics) [13] in the broader social ecology are important to consider. Bronfenbrenner’s socioecological framework [18] is a promising scaffold through which to model risk and protective factors for suicide. Latent profile analysis and latent class analysis (LPA/LCA) are well-suited as an analytic accompaniment to this framework. LPA/LCA are “person-centered” data analysis approaches which leverage variability within a sample to classify configural classes of personal and environmental attributes [19]. Recent studies harnessing LPA/LCA to model SI have been applied successfully towards predicting suicide attempts longitudinally [20] This research has primarily used psychosocial risk factors (e.g., substance use, impulsivity, hopelessness) to specify classes and have consistently provided support for a multi-class solution (≥ 3 classes) [21, 22]. However, LPA/LCA studies with adolescents [20, 23] suggest that also including protective factors (e.g., positive relationship quality, job satisfaction) can enhance class specification. To date, only one known study has examined determinants of SI using LPA/LCA among adolescents in India and found that adolescents in profiles characterized by high levels of child abuse had increased odds of self-injurious thoughts and behaviors [24].

Using a large longitudinal dataset, we selected social determinants gleaned from the past decade of research and populated them across all levels of the socioecological framework to explore constellations of social determinants–i.e., *both risk and protective factors*–with a high-risk but understudied population [14].

### Objectives

Our objectives are to (1) extract a multi-latent-class solution delineated by classes of risk and protection across levels of the social ecology, and (2) predict risk of SI, in adolescent girls from India at three-year follow-up. We hypothesize that membership in classes with heightened risk factors (e.g., higher poverty, substance use, domestic violence) will significantly increase the odds of SI at follow-up.

## Methods

### Study design

We used data from the Understanding the Lives of Adolescents and Young Adults in Bihar and Uttar Pradesh (UDAYA) study. The UDAYA study sampled 69,550 households randomly and contacted inhabitants for study participation in Bihar and Uttar Pradesh. These states are highly populous, economically weaker than other Indian states, and have low rates of female literacy [25]. Stratified multistage systematic sampling was used to identify participants. Trained interviewers administered questionnaires verbally to participants; at Wave 1, data was collected from September 2015 to January 2016 in Uttar Pradesh and from January 2016 to July 2016 in Bihar. As the study was designed to establish trends in Indian adolescent development, follow-up was undertaken three years later to shed light on factors related to transitions to adulthood. Data collection at Wave 2 occurred from October 2018 to July 2019 in both states.

A household-level questionnaire was used to interview the head of household or an adult member of the household, and an individual-level questionnaire was used to interview each adolescent in the household. The household-level data was used to derive information about the household’s wealth, caste status, religious affiliation, and geographical place of residence. The individual-level data contains information about the adolescent’s self-reported age, level of education, violence exposures, substance use, and mental health. Efforts were made by interviewers to ensure the privacy of participants by holding interviews outside the home when possible (e.g., in nearby fields), and terminating the interview without asking sensitive questions in the event that privacy was not obtainable. Survey instruments were developed to only include age-appropriate questions for young adolescents, and interviewers for this study underwent extensive ethical training in obtaining informed consent, communicating voluntary participation (and the ability to refuse to answer questions), and how to inquire about sensitive topics. Study developers also mindfully structured the interview questionnaires such that the most sensitive questions were administered in the middle of the interview, allowing for the development of rapport prior to inquiring about sensitive material, and to ensure that privacy had been established by this point in the interview. Additionally, girls surveyed in this study were provided with a confidential secondary reporting mechanism through which to answer questions about sexual violence. Specifically, to respect participant confidentiality and mitigate potential for adolescent withholding of sensitive information related to sexual violence, girls were provided with a blank card and asked to indicate on the card whether they had experienced forced sex (yes or no). Cards were then deposited in a sealed envelope, and unique study identification numbers later linked these responses to the adolescent’s interview questionnaire data. Finally, study developers partnered with local mental health nonprofit organizations, and interviewers were trained to provide referrals to these organizations and crisis lines as necessary Data were accessed with permission via the Harvard Dataverse. Detailed procedures are described in full elsewhere [25].

### Ethical approvals

The UDAYA team obtained ethical approval from the Institutional Review Board of the Indian Population Council. Written informed consent was obtained from the parent/guardian of each participant under 18 years of age. The first author of this paper obtained approval from the UDAYA study team to use their data for these analyses. Data were accessed and downloaded on March 8, 2022.

### Study sample

Adolescents were included in the analyses if they identified as girls/women and completed interviews at Wave 1 and 2 (*N* = 11,864). No exclusion criteria beyond UDAYA’s protocol exclusions [25] were applied for the purposes of this study.

### Variables and measures

#### Exposures

All 16 exposure variables were measured during Wave 1. These variables were theorized to predict classes of suicide risk based on decades of prior research on suicide in India that has highlighted the imperative role of age [10, 12], income [2], marital status [1], social discrimination [7], geographical residence [9], violence and trauma [4, 5], and mental health [10, 12] in conferring risk. These variables are as follows:

*Individual factors*. We selected five variables at the individual ecology level: age, education, wealth, substance use, and depression. Girls were asked to indicate their age (in years) at the time of first survey completion, as well as completed years of education. A household wealth index was determined by original study investigators [25]. Girls were asked about their own consumption of tobacco products, alcohol, or drugs; responses on these three items were then converted into a binary composite 0 (no substance use) or 1 (some substance use). Depression was calculated as the sum of items 1 through 8 (possible range: 0–24) on the administered Patient Health Questionnaire– 9 items (PHQ-9) [26]; a measure of depressive symptom severity that has been validated globally.

*Interpersonal factors*. We selected eight variables at the interpersonal ecology level: witnessing domestic violence, experiencing child abuse, parental substance use, marital status, arranged versus love marriage, and emotional, physical, and sexual violence. Yes/no questions assessed whether girls had ever witnessed domestic violence (father beating their mother) or had been the victim of child abuse (experienced physical violence perpetrated by a parent) and if their parents consumed tobacco products, alcohol, or drugs; responses were then converted into a binary composite 0 (no parental substance use) or 1 (some parental substance use). Girls were asked if they were married, and if their parents had arranged it (‘arranged marriage’) or not (‘love marriage’). Married girls were asked about emotional and physical violence by one’s spouse. Emotional violence was defined as humiliating the girl publicly; physical violence was defined as whether the husband had ever slapped, twisted or pulled hair, pushed/shook or thrown something, kicked dragged beaten, burnt on purpose, or attacked the girl with a knife. Finally, all girls were asked whether they had ever experienced sexual violence as forced sex by any perpetrator, attempted or forced, at any time.

*Cultural factors*. We selected three variables as factors at the cultural ecology level: geographical residence (urban/rural), religion, and caste/tribe status. A caste is a fixed, socially stratified group into which an individual is born. Members of the broad grouping of general caste are at the highest level of the social hierarchy in India, followed by those belonging to other-backwards castes. Scheduled castes (formerly called ‘untouchables’) are of the lowest, and most disadvantaged, status. Due to low endorsement for several religions, we re-categorized religion as individuals endorsing 1 (*Hindu*) or 2 (*Muslim*); all other religious categories were collapsed and coded as missing. Due to low endorsement for several caste/tribe statuses, this variable was re-categorized to include only general caste, other backward caste, or scheduled caste. Each of these three categories was treated as an independent binary variable coded as a 0 (*not affiliated with caste*) or 1 (*affiliated with caste*).

#### Outcome–suicidal ideation

Suicidal ideation was assessed at Wave 2 of data collection using item 9 of the PHQ-9; “Over the last 2 weeks, how often have you been bothered by: Thoughts that you would be better off dead, or of hurting yourself?” This variable was recoded from its original continuous scale from 0 (not at all) to 3 (nearly every day) to collapse categories into 0 (ideation absent) or 1 (any ideation present).

### Data analysis

Descriptive analyses and secondary analyses (e.g., ANOVA, chi-square, logistic regression) were conducted in IBM SPSS (Version 29.0), and LCA was conducted in Mplus Version 8.5. Full-information maximum likelihood (FIML) was used to estimate missing data in Mplus. The number of classes in the LCA was estimated freely and not a priori. All indicator variables included (i.e., 16 variables) are described in detail in the ‘Exposures’ subsection. As is consistent with best practices in LCA, class solution was ascertained from a combination of model fit statistics and interpretability. Using the MIXTURE option in Mplus, we determined model fit by integrating bootstrapped likelihood ratio tests (e.g., Vuong-Lo-Mendell-Rubin likelihood ratio test; VLMR-LRT) and relative fit indices (AIC, BIC, SSBIC) [27].

Two thousand random starts were used, and 250 optimized. Evaluation of class solution fit began with one class and multiple model fit indices (VLMR-LRT, AIC, BIC, SSBIC) were evaluated to increase the number of classes until the indices indicated an optimal fit. For each model solution tested, fit measures were recorded and used to identify the best approximating model for subsequent replications by applying interpretation rules for each measure. Following general rule-of-thumb, standard practice to evaluate change of -10 points in Bayesian information criteria (e.g., AIC, BIC, SABIC) as the minimum threshold for determining a k + 1 solution is superior. With respect to VLMR-LRT, we compared p-values generated in each class solution, attempting to identify the smallest number of latent classes for which the corresponding VLMR-LRT p-value for k versus k +1 was insignificant.

Once an optimal k class solution was identified, cases were sorted into classes based on their modal posterior probability. Mean estimated average posterior probabilities of individual cases belonging to each class were evaluated, optimal values being > ~0.70. Subsequently, individual variable entropy indices, or probability statistics between 0 and 1 (0 = low classification utility; 1 = higher classification utility), were evaluated to determine degree of class separation (optimal value > ~0.80). Mixing weights (i.e., class prevalence), or the proportion of each class in the population, were calculated. The proportions sum to 1.00 across the total number of classes. Finally, profile plots were produced for estimated variable distribution across classes. This included class-specific means on quantitative variables for continuous class indicators, and class-specific endorsement probabilities/distribution of categorical items conditional on latent class membership. The profile plots of variable distribution based on the optimal k class solution were interpreted in context to draw conclusions about patterning of socioecological factors among Indian girls.

Welch’s analysis of variance (non-parametric) and chi-square analyses were performed to examine between-class differences on indicator variables. Binary logistic regression examined how classes at Wave 1 predicted SI at Wave 2. Only data from girls who completed interviews at both waves was used for analyses.

## Results

### Descriptive statistics

The mean age of the girls was 16.56 years (*SD* = 2.17) and 19.94 years (*SD* = 2.20) during Waves 1 and 2 respectively. 58.4% of girls lived in rural communities, 78.7% identified as Hindu, and 59.5% were classified as belonging to an ‘other backward caste’ (OBC; the ‘middle’ status caste). Girls had completed 7.34 years of education on average (*SD* = 3.89), and 35.9% were married.

### Latent class analysis

#### Model selection

**Fig 1:**
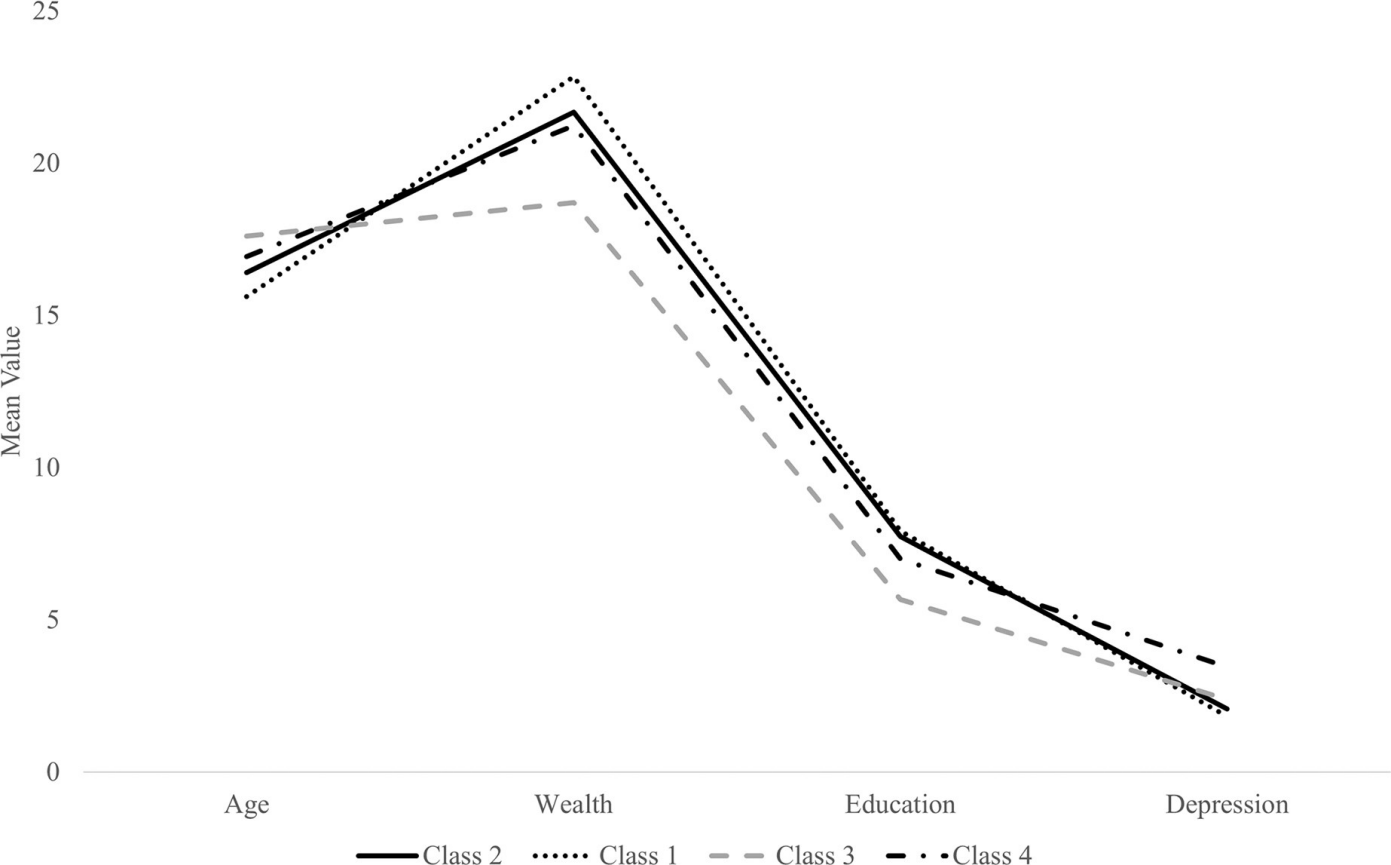
Latent four-class solution: Continuous indicators. *Note*. Class 1 = privileged class, Class 2 = modal class, Class 3 = intergenerational violence class, Class 4 = psychological distress class.

**Fig 2:**
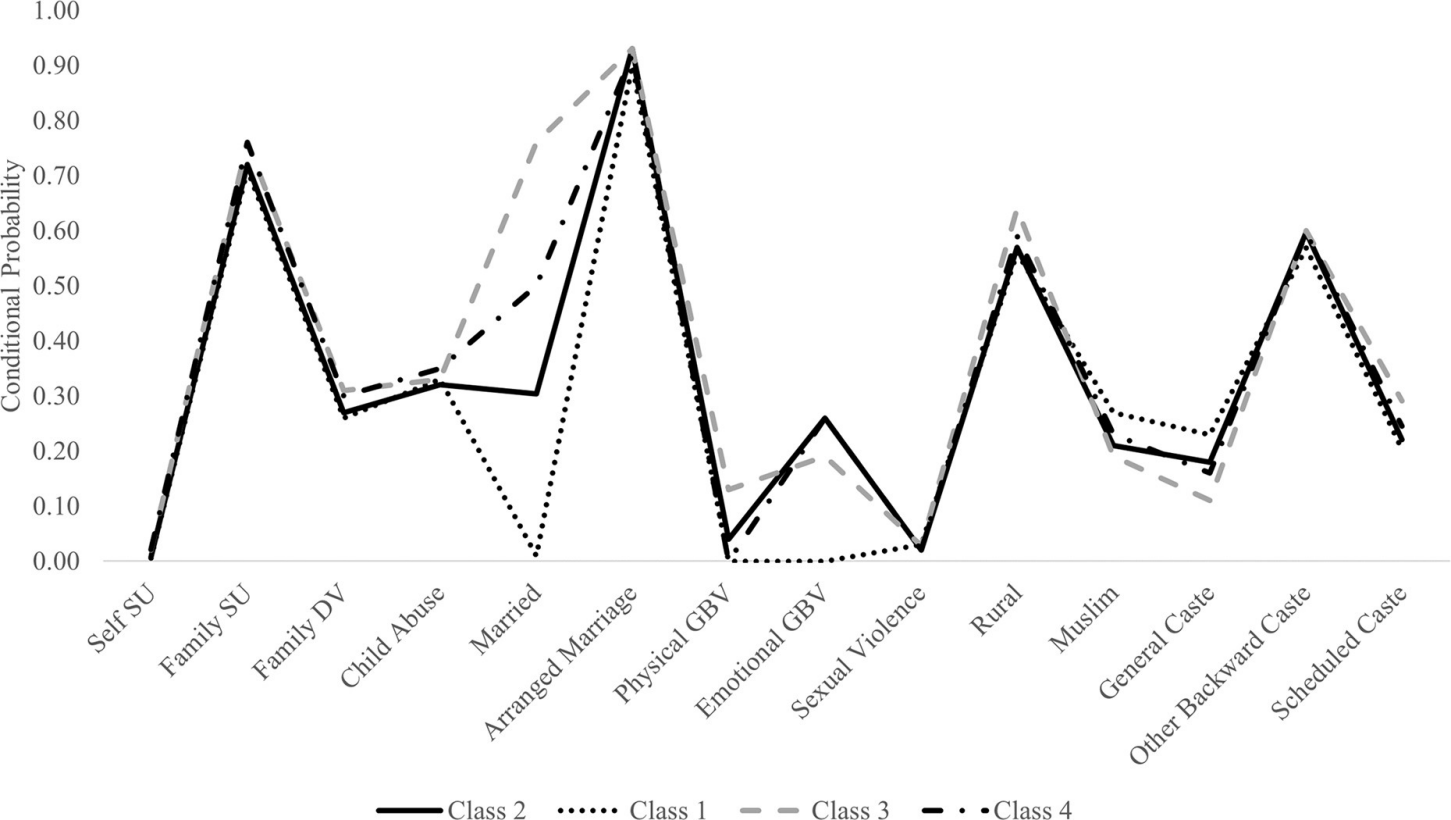
Latent four-class solution: Categorical indicators. *Note*. Class 1 = privileged class, Class 2 = modal class, Class 3 = intergenerational violence class, Class 4 = psychological distress class. SU = substance use; DV = domestic violence; GBV = gender-based violence (perpetrated by spouse).

A four-class solution was selected as the optimal model solution based on fit statistics, parsimony, and interpretability (see Figs 1 and 2). Entropy was excellent (0.89; optimal value ≥ ~0.80 (38)), and all classification probabilities exceeded 0.92 (optimal ≥.90). For detailed model fit statistics, see Table 1. Larger class solutions were investigated, but not retained due to decreasing entropy and classification probabilities that fell below .90.

**Table 1:** Fit indices.

Classes	LL	#FP	AIC	BIC	SABIC	Entropy	VLMR-LRT
One	-195568.314	21	391178.63	391333.64	391266.90	--	--
Two	-192323.47	39	384724.94	385012.81	384888.87	1.00	-195568.31***
Three	-189139.57	57	378393.14	378813.87	378632.73	.89	-192323.47***
Four	-186973.61	75	374097.22	374650.81	374412.47	.89	-189139.57***
Five	-185230.89	93	370647.78	371334.24	371038.69	.82	-186973.61***
Six	-183848.65	111	367919.30	368738.62	368385.88	.83	-185230.89***
Seven	-183042.40	129	366342.81	367294.99	366885.04	.83	-183848.65***

*Note*. LL = Log-likelihood, #FP = Number of free parameters, AIC = Akaike Information Criterion, BIC = Bayesian Information Criterion, SABIC = Sample-Size Adjusted BIC, VLMR-LRT = Vuong-Lo-Mendell-Rubin Likelihood Ratio Test

*p < .05

**p < .01

***p < .001.

#### Class classification

Findings supported a four-class solution comprised of a privileged class (Class 1; *n* = 1,470, 12.4%), a modal class (Class 2; *n* = 7549, 63.6%), an intergenerational violence class (Class 3; *n* = 2,113, 17.8%), and a psychological distress class (Class 4; *n* = 732, 6.2%). For a graphical depiction of classes, see Figs 1 and 2. Significant bivariate results are described below as between-group comparisons rather than within-group compositions; for expanded class comparisons, see Table 2.

**Table 2:** Demographic and descriptive sample properties.

Variable	Total	Class 1	Class 2	Class 3	Class 4	*Welch’s F* or χ2	*p*	Class Contrasts
	*N* = 11,864	*n* = 1,470 (12.4%)	*n* = 7,549 (63.6%)	*n* = 2,113 (17.8%)	*n* = 732 (6.2%)	*ES*: ω^2^ or φ		
	[*M* (*SD*) or n (%)]	[*M* (*SD*) or n (%)]	[*M* (*SD*) or n (%)]	[*M* (*SD*) or n (%)]	[*M* (*SD*) or n (%)]			
**Individual**								
Age (years)	16.56 (2.17)	15.62 (2.31)	16.41 (2.19)	17.60 (1.59)	16.93 (1.97)	*Welch’s F*(3, 2479) = 379.00 ω^2^ = .09	< .001	3 > all 4 > 1, 2 2 > 1
Household Wealth	21.23 (8.66)	22.84 (8.98)	21.68 (8.69)	18.70 (7.84)	21.23 (8.66)	*Welch’s F*(3, 2434) = 96.24 ω^2^ = .02	< .001	1 > all 2 > 3, 4 4 > 3
Years of Education	7.34 (3.89)	7.91 (3.48)	7.73 (3.65)	5.67 (4.45)	7.00 (3.99)	*Welch’s F*(3, 2386) = 139.24 ω^2^ = .03	< .001	2 > 3, 4 1 > 3, 4 1 = 2 3 < 4
Depression	2.19 (3.70)	1.82 (3.41)	2.08 (3.58)	2.39 (3.66)	3.45 (5.01)	*Welch’s F*(3, 2355) = 29.47 ω^2^ = .03	< .001	4 > all 3 > 1, 2 2 > 1
Self SU	139 (1.2%)	11 (0.7%)	74 (1.0%)	38 (1.8%)	16 (2.2%)	χ2(3) = 18.33 φ = .04	< .001	1 = 2 3 = 4 3, 4 > 1, 2
**Interpersonal**								
Family SU	8605 (72.5%)	1042 (70.9%)	5399 (71.5%)	1606 (76.0%)	558 (76.2%)	χ2(3) = 23.71 φ = .04	< .001	1 = 2 3 = 4 3, 4 > 1, 2
Family DV	3103 (27.7%)	355 (25.5%)	1929 (27.1%)	613 (30.7%)	206 (29.8%)	χ2(3) = 15.45 φ = .04	< .001	3 > 1, 2 1 = 2 = 4 3 = 4
Child Abuse	3843 (32.6%)	477 (32.5%)	2436 (32.4%)	679 (32.7%)	251 (34.7%)	χ2(3) = 1.58	.665	N/A
Married	4257 (35.9%)	1 (0.1%)	2277 (30.2%)	1613 (76.3%)	366 (50.0%)	χ2(3) = 2493.37 φ = .46	< .001	3 > 4 > 2 > 1
Love Marriage	326 (7.2%)	1 (10.0%)	170 (6.9%)	124 (7.5%)	31 (8.2%)	χ2(3) = 1.07	.873	N/A
Arranged Marriage	4175 (92.8%)	9 (90.0%)	2281 (93.1%)	1537 (92.5%)	348 (91.8%)	χ2(3) = 1.07	.873	N/A
Physical GBV	583 (5.1%)	0 (.0%)	287 (3.9%)	241 (12.8%)	55 (0.2%)	χ2(3) = 343.00 φ = .17	< .001	3 > 2 > 4 > 1
Emotional GBV	926 (23.2%)	0 (0.0%)	543 (25.7%)	291 (18.9%)	92 (26.4%)	χ2(3) = 25.89 φ = .08	< .001	2 = 4 2, 4 > 3 2, 3, 4 > 1
Sexual Violence	247 (2.4%)	33 (2.9%)	144 (2.2%)	56 (2.7%)	14 (2.1%)	χ2(3) = 3.70	.296	N/A
**Cultural**								
Location						χ2(3) = 38.70 φ = .06	< .001	
Urban	4934 (41.6%)	649 (44.1%)	3229 (42.8%)	754 (35.7%)	302 (41.3%)			1, 2, 4 > 3
Rural	6930 (58.4%)	821 (55.9%)	4320 (57.2%)	1359 (64.3%)	430 (58.7%)			3 > 1, 2, 4
Religion						χ2(3) = 35.52 φ = .06	< .001	
Hindu	9300 (78.7%)	1073 (73.4%)	5948 (79.1%)	1716 (81.4%)	563 (77.1%)			2 = 3 4 = all 2, 3 > 1
Muslim	2520 (21.3%)	389 (26.6%)	1572 (20.9%)	392 (18.6%)	167 (22.9%)			2 = 3 4 = all 1 > 2, 3
Caste						χ2(3) = 124.80 φ = .10	< .001	
General Caste	2044 (17.2%)	334 (22.7%)	1367 (18.1%)	226 (10.7%)	117 (16.0%)			2 = 4 1 > 2, 4 all > 3
OBC	7055 (59.5%)	837 (56.9%)	4510 (59.7%)	1272 (60.2%)	436 (59.6%)			1 = 2 = 3 = 4
SC/ST	2765 (23.3%)	299 (20.3%)	1672 (22.1%)	615 (29.1%)	179 (24.5%)			3 > 1, 2 3 = 4 1, 2 = 4

*Note*. All variables in this table refer to data collected during timepoint 1 of the UDAYA study. SU = substance use; DV = domestic violence; GBV = gender-based violence (perpetrated by spouse); OBC = other backward caste; SC/ST = scheduled caste or scheduled tribe.

Girls in the privileged class were characterized largely by high levels of protection and resources across individual, interpersonal, and cultural factors. Compared to other classes, girls in this class tended to be youngest, most educated, and most wealthy. Depressive symptom severity was lowest among girls in this class (*M* = 1.82, *SD* = 3.41). Girls in this class were least likely to be married (0.1%) and experienced the lowest rates of spousal physical violence (0.0%) and spousal emotional violence (0.0%). Girls in the privileged class were more likely than those in other classes to be of high-caste status (general caste; 22.7%) but were also the most likely to identify as members of the minority religion (Muslim; 26.6%).

The largest proportion belonged to the modal class. Girls in this class possessed levels of both risk and protective factors across socioecological levels largely demarcated as ‘moderate’ or ‘intermediate’ in comparison to other classes (which were characterized by sweeping risk or protective factors). This class possessed the second-highest levels of education and wealth; conversely, as well as second-highest rates of spousal emotional violence (25.7%).

The intergenerational violence class comprised of girls experiencing high levels of marginalization and risk across individual, interpersonal, and cultural factors. Girls in this class tended to be the oldest, the least educated, and the least wealthy. Girls witnessed domestic violence at the highest rates compared to other classes (30.7%), were the most likely to be married (76.3%) and experienced the highest rates of spousal physical violence (12.8%). Culturally, more girls in this class lived in rural locations (64.3%). While a high proportion were likely to be of low-caste status (SC/ST; 29.1%), membership in this class was also associated with the highest rates of majority religious status (Hindu; 81.4%).

Finally, girls in the psychological distress class (*n* = 732, 6.2%) were uniquely characterized by a pattern of psychological variables (primarily interpersonal) to include depressive symptoms, substance use, and emotional violence inflicted by a spouse. Specifically, in comparison to other profiles, girls in the psychological distress class were most likely to endorse high depressive symptomatology (*M* = 3.45, *SD* = 5.01). This class indicated relatively high levels of parental (76.2%) substance use, and though reports of self-substance use in this sample were low overall, the girls in this class were the most likely of the four classes to report substance use (2.2%). Of the classes, girls in this group were second most likely to be married (50%) and reported high rates of spousal emotional violence (26.4%). Additionally, although class comparisons were non-significant, girls in the psychological distress class were most likely (34.7%) to endorse having experienced child abuse.

### Class membership as a predictor of suicidal ideation

At three-year follow-up, binary logistic regression (χ^2^ = 33.47, *df* = 3, *p* < .001) with a sensitivity rate of 91% found that compared to the privileged class, membership in the modal class was associated with a 33% increased risk of current SI (OR: 1.33, 95% CI:[1.08, 1.64]), membership in the intergenerational violence class was associated with a 79% increase in SI (OR: 1.79, 95% CI:[1.42, 2.26]), and membership in the psychological distress class was associated with a 84% increase in current SI (OR: 1.84, 95% CI:[1.38, 2.47]). See Table 3 for more detailed regression model results.

**Table 3:** Odds ratios and 95% confidence intervals for predictors of suicidal ideation at 3-year follow-up.

Predictor	*B*	*SE*	OR	95% CI	*p*
^a^ Privileged Class	--	--	1.00	--	--
Modal Class	0.29	0.11	1.33	1.08–1.64	.007
Intergenerational Violence Class	0.58	0.12	1.79	1.42–2.26	< .001
Psychological Distress Class	0.61	0.15	1.84	1.38–2.47	< .001

*Note*. B = unstandardized beta value; SE = standard error; OR = odds ratio; CI = confidence interval.

^a^The reference class for interpretation of odds ratio.

## Discussion

Our study is the first to examine intersectional identities and syndemics across all levels of the social ecology and their longitudinal relationship with SI in a large representative cohort of Indian girls. Our multi-class solution identified four unique risk classes using intersectional identities and diverse social determinants. Nearly two thirds of the sample fit into Class 2 –defined by a mixture of risk and protective factors–suggesting that this confluence of factors is most typical for North Indian girls. The remaining profiles were characterized by 1) relative privilege (Class 1; more protective than risk factors), 2) high intergenerational violence (Class 3; higher domestic violence), and 3) high psychological distress (Class 4; higher rates of depression, substance use, and emotional violence). Notably, the confluence of risk factors in all classes significantly heightened the risk of SI at follow-up. The strongest confluence of risk factors emerged among girls with high psychological distress (Class 4), such that these girls had an 84% greater likelihood of SI at three-year follow-up, highlighting the need to enhance mental health for effective suicide prevention.

Our study illustrates how intersectional identities interact with broader syndemics across the social ecology. As seen in classes 1 and 3, socioeconomic status factors tend to cluster (e.g., wealth, education) and confer either broad risk or broad protection. Similarly, broader cultural factors (e.g., geography, caste) also co-occur. Girls in Class 3 came from lower caste backgrounds and rural areas, which confers social disadvantage and increases other exposures such as marrying young and spousal violence [1, 2]. At three-year-follow-up, this class evinced the second-highest risk of SI, illustrating compounding social disadvantages across the individual and interpersonal domains of the social ecology. Conversely, girls in Class 1 (the ‘privileged’ class) showed the opposite pattern: these girls were born into relatively better cultural circumstances, which conferred advantage and protection that self-perpetuated and resulted in lower SI risk 3 years later. This finding is consistent with studies documenting how women owning property–a proxy for greater wealth equity–buffers against violence after marriage [3]. Overall, cultural factors set the tone for one’s overall social advantage and these factors interact with individual and interpersonal factors in unique ways.

Membership in a class with a high proportion of marriage (i.e., Classes 3, 4) was associated with increased SI risk at follow-up. This finding is consistent marriage serving as a risk factor because marriage increases opportunity for GBV, which is highly linked with SI [28]. Similarly, membership in a class where girls endorsed higher rates of substance use and depression is consistent with the literature that this constellation of factors increases SI risk [4]. Reinforcing cycles of depression and substance use may serve to self-medicate against distress in the short-term, but as demonstrated in the current study, is likely to increase likelihood of worse functional outcomes and heightened psychological distress (i.e., SI) long-term.

We also detected no significant between-class differences on marriage type (arranged/love] and rates of child abuse and sexual violence. Research indicates that rates of child abuse and sexual violence for girls in India may largely transcend socioeconomic and cultural strata as culturally assumed norms, with scarce criminalization of such violence [5]. These exposures were evenly distributed across our profiles, which suggests that these three factors were invariable, even as individual, interpersonal, and cultural profiles differed [29]. Additionally, consistent with our findings, upwards of 90% [30] of all marriages in India are arranged—this prevalence tends to be consistent across sociodemographic groups, as within-caste marriage is of high cultural importance.

### Strengths and limitations

Our study is limited in a few important ways. First, SI is a fluctuant phenomenon rather than a relatively stable disease category such as depression; ideally, our study would account for the frequency and severity of SI but these data were not collected between Wave 1 and Wave 2 and our study is a secondary analyses of the original UDAYA study design. As SI is fluctuant, future studies that use agile methods of assessment (e.g., daily digital diaries) in conjunction with profiling can provide more nuanced information on the frequency and severity of SI [31]. Second, we had a narrow range of ages in this study, which could cause range restriction. Future studies should recruit early adolescent girls to model risk during this critical window. Additionally, using an interview format to collect data may have caused discomfort for the interviewer and girls on highly stigmatized topics, which could have led to demand characteristics and/or under-reporting certain exposures. Additional research can employ multi-method data collection to reduce bias.

Our study also has several strengths. We used a large sample size of a high-risk population, increasing confidence in our findings with diverse subgroups. Further, we integrated individual-level risk factors with social determinants (i.e., intersectional identities *and* syndemics) to model a theoretically driven LCA. The religion and caste affiliations in our sample closely mimic estimates of the sociodemographic composition of northern Indian states [25], speaking to the generalizability of our findings. This work augments an evidence base where few studies have explored risk and protective factors *across time* to make causal predictions; by combining LCA and logistic regression, we use a stronger approach to establishing which social determinants at baseline are most crucial to prioritize for suicide prevention later.

### Conclusions and implications for suicide prevention

This study offers a unique contribution to the fields of suicide prevention and public health by using a systems-approach to modeling a comprehensive set of factors encompassing one’s intersectional identities and broader syndemics to predict SI. Our study reinforces the need for early intervention for improved psychological, health, and social outcomes. A strong public health response includes both targeted screening and indicated interventions. This two-pronged strategy will improve suicide prevention when directed at young girls from rural India with less wealth, from a lower caste, and from households where violence and substance use co-occur. Finally, this systems-based approach to identifying types of people to target for intervention based on constellations of risk factors can improve public health approaches for suicide–a notoriously difficult–yet preventable–phenomenon to predict.

Our study highlights important risk factors that are modifiable at the population level, including poverty. A systematic review demonstrated in 60% of LMIC, poverty is associated with increased fatal and non-fatal suicide outcomes [2], making economic disadvantage a promising intervention target among young Indian girls. However, poverty is complex and systemic; consequently, addressing poverty in suicide prevention is likely to require maintaining a systems approach to understanding direct and indirect influence of key social determinants (e.g., education, healthcare, trauma exposures [2]) on SI [2]. At the individual level, we need a public health response with better screening and timely intervention delivery. Care for depression, violence prevention, and substance use remain particularly essential given our results. We recommend regular suicide risk assessments and linkage to services for groups of girls with limited access to education and financial resources, who reside in rural locations, and have a low-caste background. Although India has major treatment gaps due to a lack of mental health infrastructure, psychosocial assessments and interventions can be delivered effectively by non-specialists and peers in LMIC [32].

Additionally, breaking cycles of intergenerational violence is a high-need area. These girls (Class 3) evinced the second greatest risk of SI at follow-up. Witnessing parental violence and experiencing emotional violence predicts teen dating violence, thereby increasing the risk of future sexual and physical violence [33]. Domestic violence awareness campaigns and deploying maternal care workers to deliver parenting skills training and improve parental mental health might improve youth outcomes.
